# Low-Energy Shape Resonances of a Nucleobase in Water

**DOI:** 10.1021/jacs.2c11440

**Published:** 2022-12-30

**Authors:** Graham A. Cooper, Connor J. Clarke, Jan R. R. Verlet

**Affiliations:** Department of Chemistry, Durham University, Durham DH1 3LE, U.K.

## Abstract

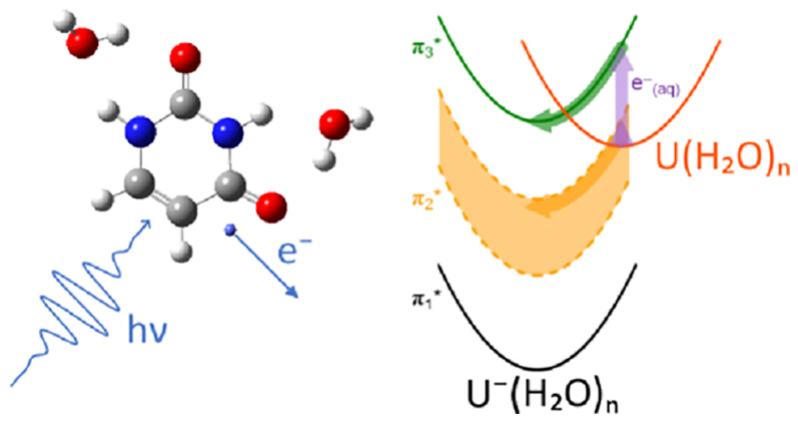

When high-energy radiation passes through aqueous material, low-energy electrons are produced which cause DNA damage. Electronic states of anionic nucleobases have been suggested as an entrance channel to capture the electron. However, identifying these electronic resonances have been restricted to gas-phase electron-nucleobase studies and offer limited insight into the resonances available within the aqueous environment of DNA. Here, resonance and detachment energies of the micro-hydrated uracil pyrimidine nucleobase anion are determined by two-dimensional photoelectron spectroscopy and are shown to extrapolate linearly with cluster size. This extrapolation allows the corresponding resonance and detachment energies to be determined for uracil in aqueous solution as well as the reorganization energy associated with electron capture. Two shape resonances are clearly identified that can capture low-energy electrons and subsequently form the radical anion by solvent stabilization and internal conversion to the ground electronic state. The resonances and their dynamics probed here are the nucleobase-centered doorway states for low-energy electron capture and damage in DNA.

## Introduction

When high-energy radiation passes through aqueous media, including within living organisms, low-energy (<20 eV) electrons are produced.^1^ These chemically active species have been shown to directly cause DNA damage through single and double-strand breaks that occur at distinct electron energies.^2,3^ The first step in this process is the transient electron capture through electronic resonances of DNA components. The anion resonances of the nucleobases, in particular, have been extensively studied through isolated electron-nucleobase reactions.^4−10^ These have clearly identified the resonances and dissociative electron attachment pathways that exist, and have offered detailed mechanistic insight into the chemical dynamics involved in the isolated nucleobase. However, how these observations relate to the aqueous environment of DNA has remained elusive.^11^ Linking the bottom-up gas-phase work to top-down condensed-phase studies would provide unprecedented insight into the primary processes in DNA. To bridge these two approaches, complexity can be added incrementally to gas-phase nucleobases using, for example, nucleobase-solvent clusters. However, this has been challenging in electron-spectroscopic experiments because of the lack of mass-selection prior to the experiment.^12,13^ To overcome this challenge, we have shown that 2D anion photoelectron spectroscopy can probe electron-molecule resonances following optical excitation, offering new and complementary information to electron spectroscopy.^14,15^ More importantly, mass-selection offers a route to probing the effect of incremental solvation on electronic resonances.^16,17^ We now apply this methodology to a radical nucleobase anion clustered with water molecules and uncover how the electron impact resonances evolve with increasing hydration, thus identifying the possible resonances and their dynamics responsible for nucleobase electron capture that constitutes the first step of low-energy electron damage to DNA.

We focus on uracil, U, the simplest of nucleobases and a proxy for pyrimidines due to their similar electronic resonances.^4^ Furthermore, the effect of micro-hydration of pyrimidine resonances has been considered by computational methods.^18−23^ The isolated uracil radical anion, U^–^, is a dipole-bound anion in the gas-phase, where the excess electron is very weakly bound by the dipole-moment of the molecular core of U.^24^ Solvation substantially stabilizes the valence anionic states and a single water molecule is sufficient to render the valence state (π_1_*) as the lowest energy conformation.^25^ The stabilization has been studied using photoelectron spectroscopy for uracil-water clusters, U^–^(H_2_O)_*n*_, with *n* ≤ 7, which showed that increased hydration further stabilizes the valence anion.^26,27^ However, extrapolation from such small *n* to larger sizes and the bulk is inappropriate. Hence, key physical quantities such as the vertical detachment energy, VDE, and adiabatic electron affinity, AEA, of the aqueous uracil anion, U^–^_(aq)_, are not known. Moreover, because previous experiments focused on a few distinct photon energies, they offered no insight into the location or dynamics of resonances that are key to the radiation damage mechanism. Experiments by Kočišek et al. have measured the anionic products formed following electron impact (<3 eV) of small U(H_2_O)_*n*_ (and thymine-water) clusters and found that the gas-phase dissociative electron attachment channel to produce [U – H]^−^ is suppressed in the cluster.^12^ Here, we study U^–^(H_2_O)_*n*_ with *n* up to 10 to understand the evolution of electronic resonances with increased hydration, and we measure additional photoelectron spectra up to *n* = 35 to determine the evolution of detachment energies. Together, these offer a detailed view of the energetics of electronic states in aqueous solution that ties gas-phase work to the bulk.

## Experimental Methods

The experiment has been described in detail previously.^28^ A sample of uracil (Sigma Aldrich, ≥99%) was heated to a temperature of 230 °C in the oven of a high-temperature Even-Lavie valve.^24^ Nitrogen gas (Oxygen-Free grade) at ∼5–6 bar was passed through this reservoir and expanded into vacuum to produce a seeded supersonic expansion.

Uracil radical anions were produced using a ring-filament ionizer, which consisted of a thoriated tungsten wire filament (Goodfellow, 1% Th, 0.25 mm diameter) carrying a ∼5 A current. The filament was contained in a hollow stainless steel cylinder (O.D. 55 mm, thickness 7 mm) with a ∼15 mm hole through the center and held at between −200 and −600 V (tuned to optimize anion production) to serve as an anode, which, combined with a grounded grid around the central hole, accelerated the electrons produced by the filament towards the supersonic expansion that passed through the center of the hole.

The electron impact on the seeded gas jet produces a variety of ionic and neutral species, including anions, which were extracted using a perpendicular time-of-flight mass spectrometer. A representative mass spectrum is shown in Figure S1. Packets of mass-selected ions were subjected at the focal point of the spectrometer to light from a Nd:YAG pumped optical parametric oscillator (Continuum, Horizon), producing electrons which were collected using a velocity map imaging photoelectron spectrometer.^29^ The images were reconstructed using polar onion peeling^30^ and calibrated using iodide. The resultant energy resolution was on the order of 5% of the photoelectron’s kinetic energy.

## Results and Analysis

**Figure 1:**
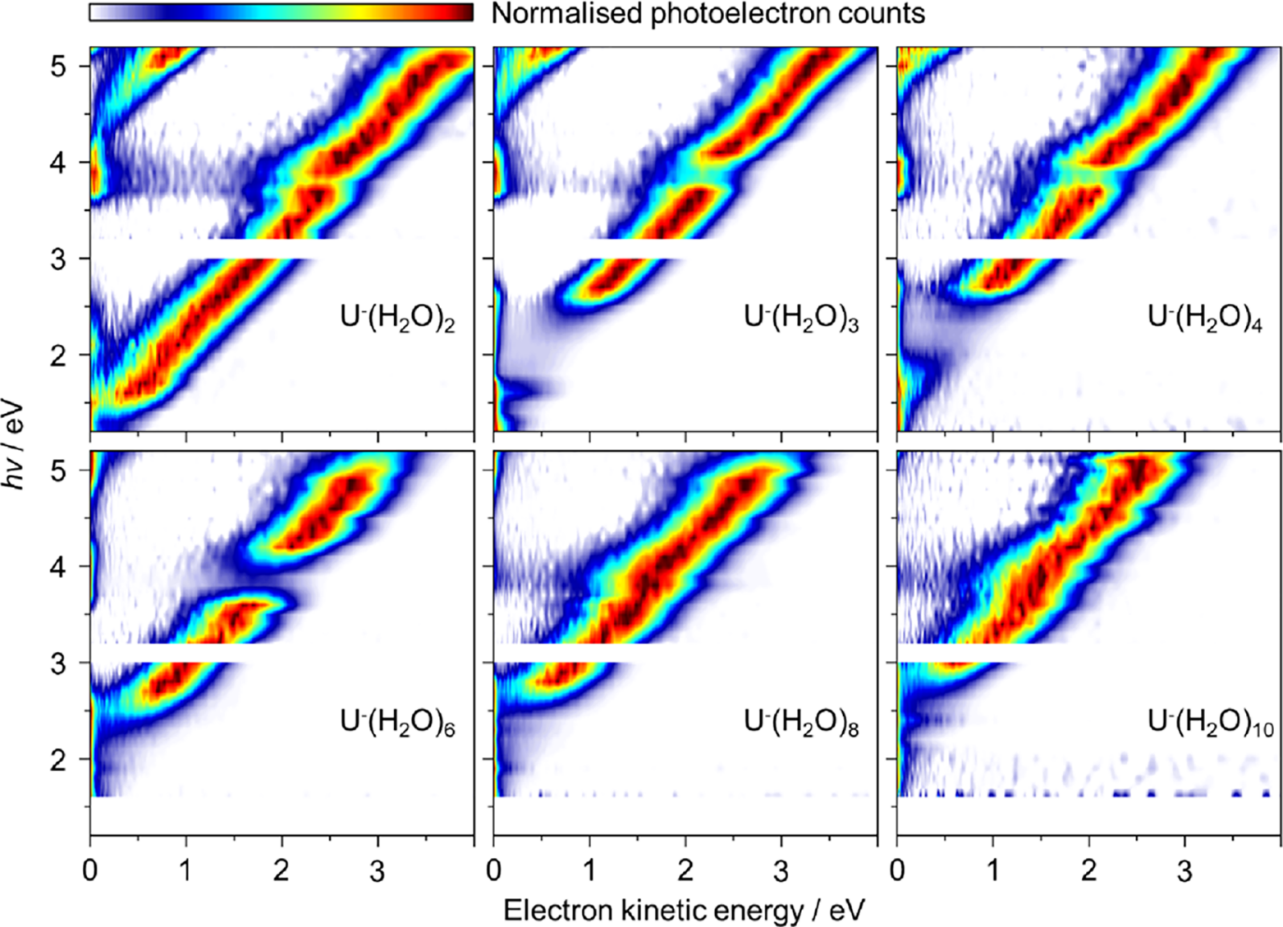
2D photoelectron spectra for U^–^(H_2_O)_*n*_ with *n* = 2–4, 6, 8, and 10. Individual photoelectron spectra were recorded at 0.1 eV intervals and normalized to their maximum intensity. The spectrum at *hv* = 3.1 eV was not collected.

Cold anionic clusters of U^–^(H_2_O)_*n*_ were produced and mass-selected before being intersected by nanosecond light pulses with photon energies, *hv*, ranging from 1.2 ≤ *hv* ≤ 5.2 eV. Figure 1 shows the 2D photoelectron spectra of U^–^(H_2_O)_*n*_ with *n* = 2–4, 6, 8, and 10. Each 2D plot is composed of several individual photoelectron spectra at 0.1 eV *hv* intervals that have been normalized to their maximum intensity (the *hv* = 3.1 eV spectrum is omitted because of weak output from the optical parametric oscillator at 400 nm).

Figure 1 shows features and trends that appear common to all clusters. Firstly, the dominant diagonal feature corresponds to direct detachment from the D_0_ anionic ground state of U^–^(H_2_O)_*n*_ to the S_0_ ground state of the corresponding neutral cluster, and is in agreement with previous studies at specific photon energies.^26,27^ The electron kinetic energy (eKE) increases linearly with *hv* with a unit gradient, as expected for such a process. As *n* increases, the onset of the direct detachment feature moves to higher *hv* indicating a larger electron binding energy (eBE = *hv* – eKE) for larger *n*. For *n* = 2 and 3, a second, parallel feature is seen in the top-left corner, which corresponds to detachment to the lowest excited triplet state of the neutral with a S_0_-T_1_ gap of ∼3 eV. The onset of this is also visible for *n* = 4.

**Figure 2:**
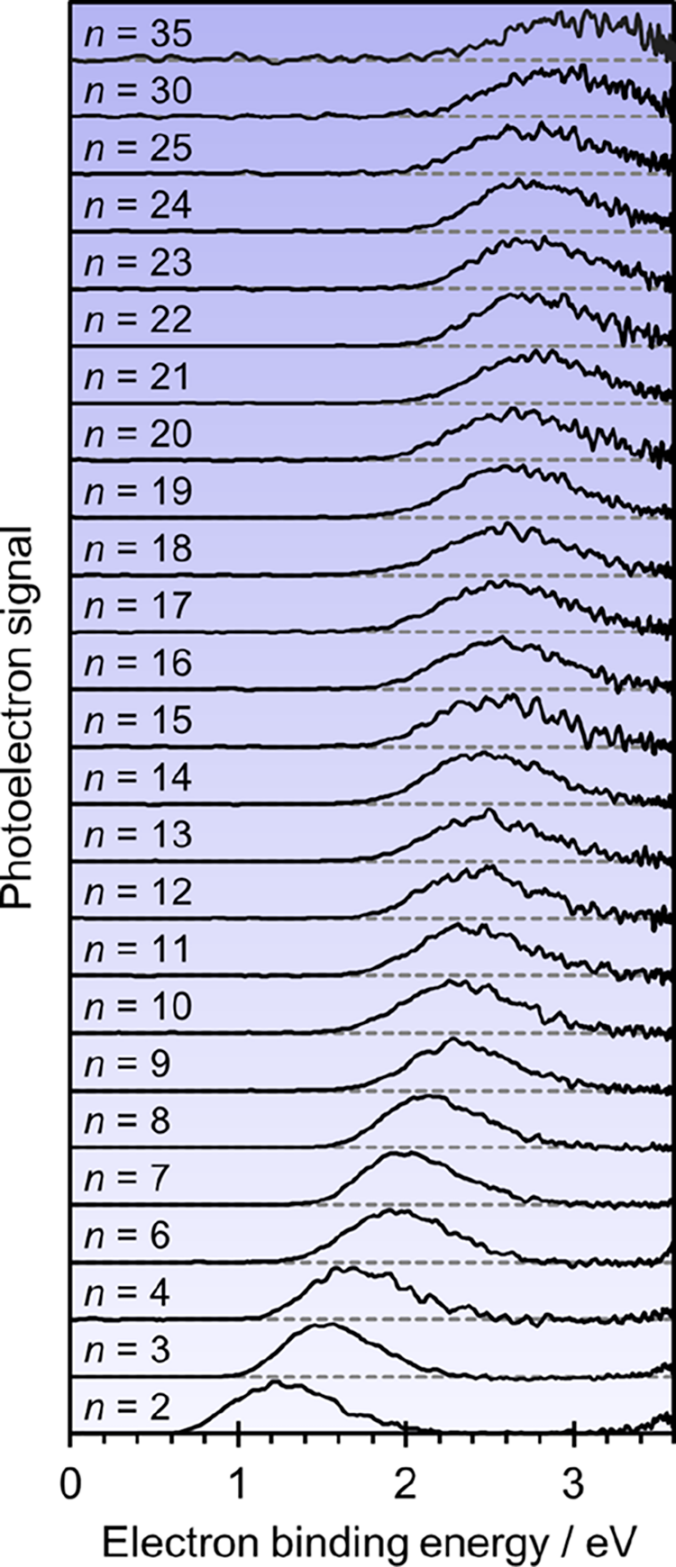
Photoelectron spectra recorded at *hv* = 3.6 eV for U^–^(H_2_O)_*n*_ clusters. Spectra are offset for clarity and normalized to their peak intensity.

For clusters with *n* > 10, 2D photoelectron spectroscopy becomes prohibitively time-consuming and we instead measured their photoelectron spectra at a single photon energy, *hv* = 3.6 eV. These photoelectron spectra are shown in Figure 2. As *n* increases further, so does the binding energy, but at a slower rate than for the smaller clusters. Additionally, the spectral width increases with increasing *n*.

**Figure 3:**
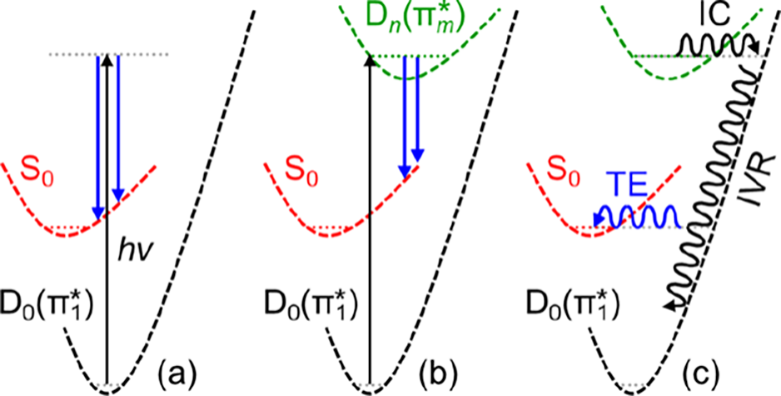
Schematic of possible electron emission processes (blue downward arrows) following excitation (black upward arrow) of U^–^(H_2_O)_*n*_ clusters from the D_0_(π_1_*) ground state. (a) Direct (non-resonant) detachment into the continuum. (b) Autodetachment from excited anionic states (resonances), D_*n*_(π_m_*), where the electron kinetic energy (length of blue arrow) differs from (a). (c) Thermionic (statistical) emission from the ground electronic anionic state, which is populated through internal conversion (IC) and equilibrated through internal vibrational redistribution (IVR).

In addition to the direct photodetachment channel (Figure 3a), electronic resonances can be photoexcited. Evidence for two separate anion resonances can be seen in Figure 1 as both a reduction in eKE of the direct detachment peak, and as an apparent depletion in intensity because photoelectron signal at very low eKE becomes dominant. These changes arise because of the competition between direct and indirect electron detachment.^15^ Indirect detachment can take two forms. Electrons can be emitted from the resonance by autodetachment, leading to changes in eKE and broadening of signal in the 2D photoelectron spectra (Figure 3b).^15,31−34^ Alternatively, electron loss can occur through a statistical process from the bound anionic ground state that has been repopulated by internal conversion from the electronic resonance (Figure 3c).^14,35,36^ Such thermionic emission leads to very low energy electrons with a characteristic Boltzmann distribution.^37−39^ Both thermionic emission and shifts in eKE are apparent in specific regions of Figure 1 and are direct evidence of resonances being excited.^15^ Two resonances are clearly observed: one is a broad feature observed between *hv* ∼ 1.5–2.7 eV; and the second is a much sharper feature centered around *hv* ∼ 3.9 eV. These can be correlated to the π_2_* and π_3_* shape resonances of bare U^–^, which have been observed by electron transmission spectroscopy at electron impact energies of 1.58 and 3.83 eV, respectively.^4^ At *hv* > 5 eV, evidence of a further resonance can be seen in some clusters.

**Figure 4:**
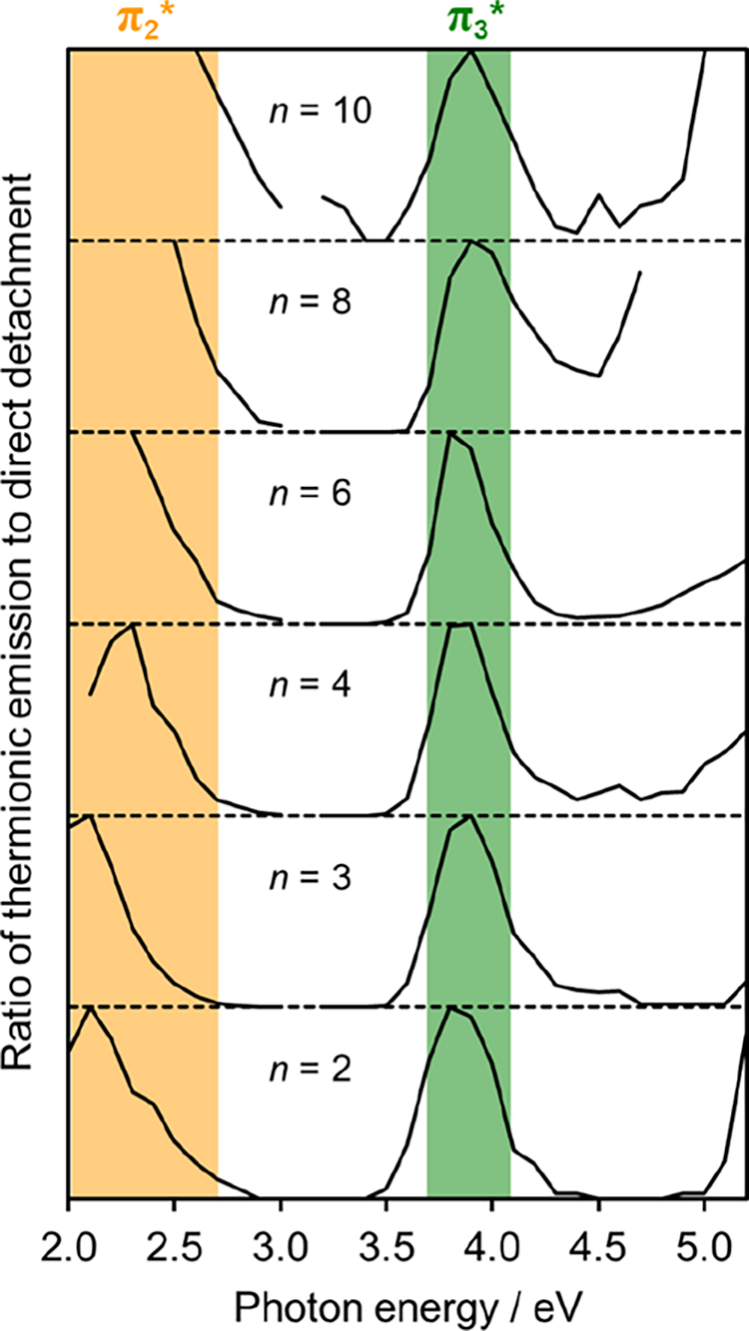
Ratio of thermionic emission to direct detachment as a function of photon energy, *hv*, for various U^–^(H_2_O)_*n*_ clusters. Graphs are offset for clarity. Orange and green shaded regions show the range over which the π_2_* and π_3_* resonance is excited, respectively. The ratios have been normalized to peaks in the range *hv* < 3.1 eV and 3.1 < *hv* < 4.5 eV for clarity.

To determine the position of the resonances more precisely, the relative contribution of thermionic emission at each *hv* was determined by fitting each spectrum to a sum of an exponentially decaying function (representing thermionic emission) and a Gaussian function (representing direct detachment) and then taking the ratio of their respective amplitudes (see Figure S2). The result of this analysis is shown in Figure 4, where peaks have been normalized to aid comparison between clusters. The ratio provides a reasonable estimate of the relative excitation cross-section (and hence resonance energetic location) under the assumption that the population of the resonance decays by thermionic emission.^32^ There will also be contributions from direct detachment channels at *hv* close to their onset and these have been omitted. The observation of distinct peaks in Figure 4 is clear evidence that these correspond to resonances of U^–^ and not to electron transfer to the water cluster. Specifically, the fact that peaks do not shift with hydration is consistent with previous 2D photoelectron spectroscopy studies on micro-hydrated polycyclic aromatic hydrocarbon anions.^16,17^ Additionally, the peaks in Figure 4 align with the electron impact resonances of the isolated uracil molecule once stabilization of the anion through incremental hydration is considered. Nevertheless, photoexcitation could trigger charge transfer to solvent that subsequently leads to thermionic emission, but such excitations are limited in *hv* range to just below the VDE^40−43^ and this range has been omitted in Figure 4 to exclude its possible contribution.

Figure 4 reveals two separate resonances. The peak around *hv* ∼ 3.9 eV corresponds to excitation of π_3_* and results in a relatively sharp feature that is present for all six clusters studied. The spectral profile of the π_3_* resonance was fitted to a Gaussian function for each *n* and yields an average value for its center of *hv* = 3.89 ± 0.03 eV and a full-width at half-maximum of 0.34 eV.

A second region of enhanced thermionic electron emission in Figure 4 corresponds to excitation to the π_2_* resonance, although it is less clearly defined. The thermionic emission signal is present even at the lowest photon energies used and even below the VDE, such that we cannot determine the lower bound of the resonance. For the clusters studied, the relative intensity changes significantly with increasing *hv*, but for all clusters shows a clear decline at ∼2.7 eV, which corresponds to the upper bound on exciting the π_2_* resonance. As such, the *hv* region marked in orange in Figure 4 that can be assigned to the π_2_* resonance is <1.5–2.7 eV. Its spectral width of more than 1 eV in hydrated clusters is similar to that seen for electron attachment in the isolated nucleobase.^7^

The observation of thermionic emission is consistent with the results of Kočišek et al., who observed intact U^–^(H_2_O)_*m*_ clusters upon electron impact to U(H_2_O)_*n*_ (where *m* ≤ *n*) instead of the formation of [U – H]^−^ by dissociative electron attachment as seen for bare U.^12^ While their experiment would not be sensitive to anions decaying by thermionic emission, their observation of U^–^(H_2_O)_*m*_ also necessitates the formation of ground state anions, where stabilization is induced by the dissipation of energy into the many modes of the cluster and/or H_2_O evaporation. Hence, the current experiments are complementary by probing the competing electron evaporation channel, whilst importantly offering mass-selectivity. For *n* = 8 and 10, the contribution of thermionic emission from the π_3_* resonance diminishes (see Figure 1), presumably because there are many more internal modes and because the electron affinity has increased to such an extent that thermionic emission becomes less favorable (or slower) than H_2_O evaporation.

**Figure 5:**
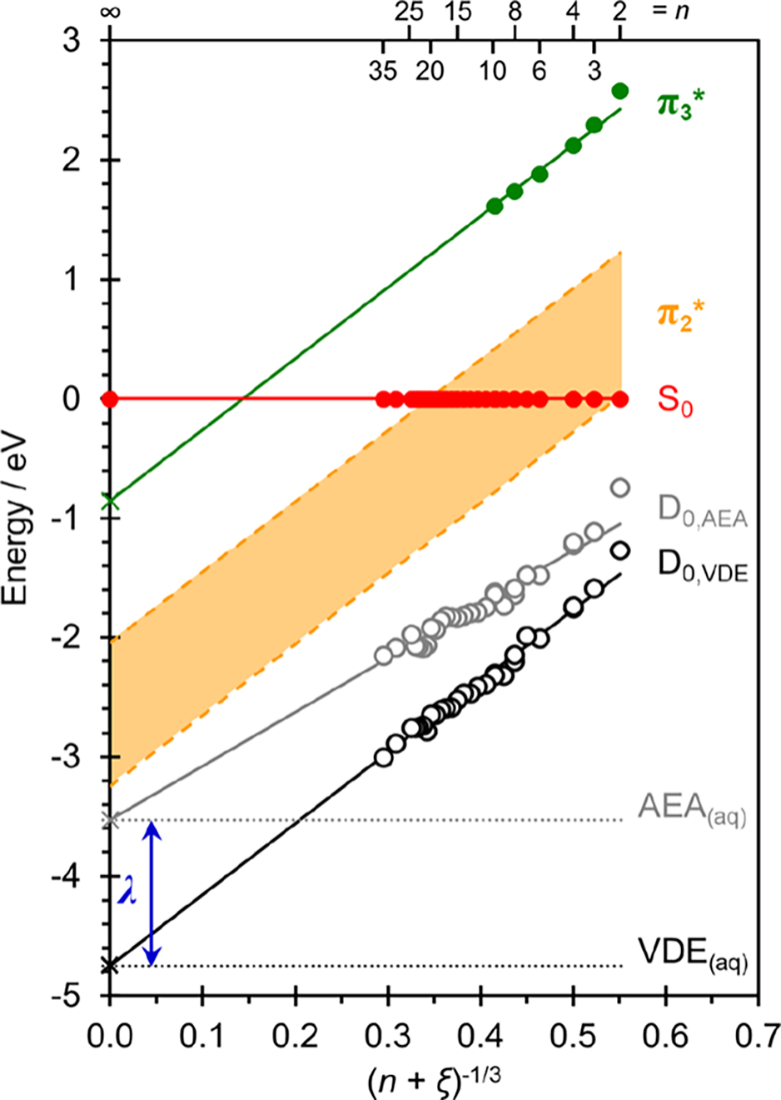
Energy level diagram for U^–^(H_2_O)_*n*_ clusters, relative to the ground state of the neutral cluster, S_0_, plotted as a function of cluster size, (*n* + ξ)^−1/3^. Circles represent data taken from Figures 2 and 4 for the D_0_ ground state and the π_3_* resonance, and the dashed lines represent the extracted range of the π_2_* resonance. Linear fits (considering *n* ≥ 3) to the vertical detachment energy, VDE, and adiabatic electron affinity, AEA, are extended to their respective bulk aqueous limits, shown with crosses. The difference between these extrapolated energies, VDE_(aq)_ and AEA_(aq)_, corresponds to the reorganization energy, λ. The gradients of the lines fitting the π_2_* and π_3_* resonances were matched to that of the VDE.

In addition to the resonance positions, we also determine the VDE of U^–^(H_2_O)_*n*_ from Figures 1 and 2 by fitting the direct detachment peak to Gaussian functions for non-resonant wavelengths (Figure S3). The adiabatic electron affinity, AEA, for each cluster is estimated by considering the onset of each peak (taken as 10% of peak height, Figure S3). The error in determining the VDE is on the order of ±0.05 eV. Using the VDE, AEA, and electronic resonance positions for each *n*, an energy level diagram for U^–^(H_2_O)_*n*_ can be constructed, as shown in Figure 5. The energy is referenced to the neutral S_0_ ground state (defined as 0 eV).

For sufficiently large values of *n*, physical properties can be correlated to the cluster’s size and evolve linearly as a function of *n*^–1/3^.^44,45^ Such a correlation appears to fit the data very well for U^–^(H_2_O)_*n*<35_. However, the gradient of VDE as a function of *n*^–1/3^ does not agree with that expected from continuum dielectric theory in the bulk limit.^46,45^ This is predominantly because of a disregard for the volume of the uracil anion in the cluster. To account for this missing volume, the energy level diagram in Figure 5 is plotted against (*n* + ξ)^−1/3^, where ξ represents the volume occupied by the uracil anion relative to the volume a single water molecule, assuming a bulk solvated U^–^. A value of ξ = 4 produces a gradient in agreement with the dielectric sphere model (whether U^–^ is surface or internally solvated).^47^ This choice of ξ is supported by considering the relative molar volumes of U^–^ and H_2_O as well as electronic structure calculations (see Supporting Information for details),^48^ and the resulting gradient is also consistent with other anion-water cluster studies.^49,50^ The linear trend is expected for sufficiently large clusters, while small clusters may be expected to deviate. In Figure 5, the smallest cluster (*n* = 2) shows the greatest deviation to the linear trend and is not incorporated into the linear fits. Extrapolation of VDE as a function of (*n* + ξ)^−1/3^ to the bulk limit at *n* = ∞ (i.e., (*n* + ξ)^−1/3^ = 0) gives a VDE of U^–^ in aqueous solution, VDE_(aq)_ = 4.75 ± 0.20 eV. The error in determining VDE_(aq)_ is predominantly associated with the choice of ξ (see Supporting Information) and selecting only larger clusters for the linear fit (e.g., *n* ≥ 16, which is expected to be more representative of U^–^_(aq)_) changes VDE_(aq)_ by less than the error.

Figure 5 also includes the π_2_* and π_3_* resonances. Whilst we only have data for relatively small clusters (*n* ≤ 10), the gradient as a function of (*n* + ξ)^−1/3^ for the π_3_* resonance is similar to that of the VDE, offering confidence that the resonance energies can also be extrapolated to larger sizes. In agreement with recent studies on the effect of hydration on polycyclic aromatic hydrocarbon anions,^16,17^ the excitation energies to the resonances are invariant to cluster size, however, from the perspective of a free electron impacting the neutral (S_0_) molecule, the resonances become stabilized by an extent similar to that of the anion ground state.^16^

## Discussion

Figure 5 provides detailed insight into the energetics and resonances of the hydrated uracil radical anion, U^–^_(aq)_. Both the π_2_* and π_3_* shape resonances are bound states in U^–^_(aq)_, with the later lying ∼0.85 eV below S_0_. More generally, Figure 5 shows that electron-impact resonances can be experimentally determined for closed shell molecules in aqueous environments.

**Figure 6:**
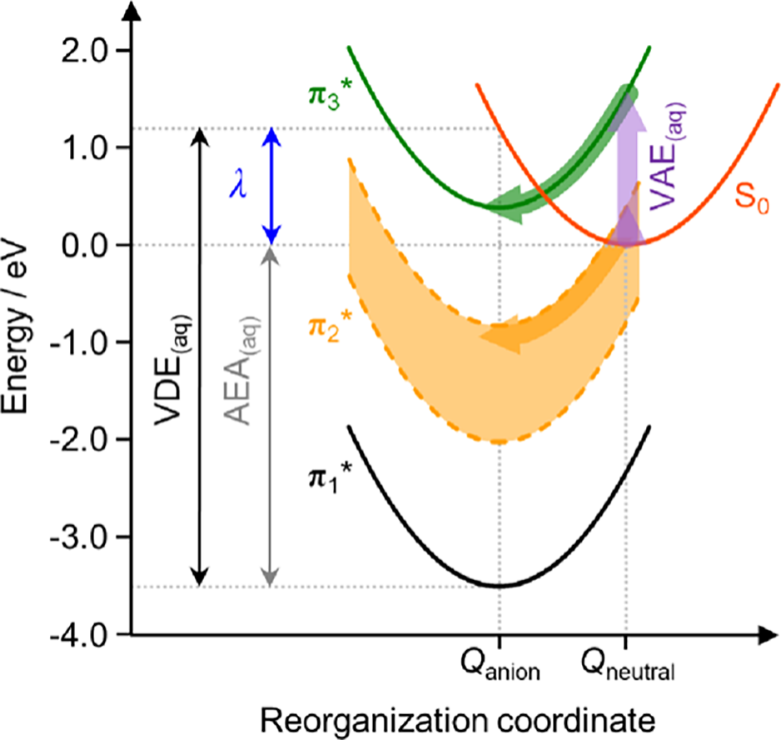
Schematic Marcus picture showing aqueous-phase energy curves, with energies determined from the extrapolation of uracil-water cluster anions. The VDE_(aq)_ and AEA_(aq)_ correspond to the aqueous vertical detachment energy and adiabatic electron affinity, respectively. The reorganization energy is defined by λ. Upwards block arrows show the vertical electron attachment to the aqueous neutral uracil, with horizontal block arrows showing the dynamical evolution following electron attachment, which stabilize the generated anion resonances through reorganization.

It is important to recognize that the current work considers the resonances within the anion’s geometry and hydration sphere. Vertical electron attachment on the other hand will lead to U^–^ in the neutral geometry and hydration sphere. The energy difference between the two is the reorganization energy, λ. An estimate of λ can be obtained from the difference in VDE_(aq)_ and AEA_(aq)_, as highlighted in the Marcus-type plot in Figure 6. From Figure 5, we find that the AEA also extrapolates linearly with (*n* + ξ)^−1/3^ to a value of AEA_(aq)_ = 3.53 ± 0.20 eV, which compares well with a recent computed prediction of 3.37 eV.^51^ Hence, from our experiment we find that λ = 1.2 eV. The diverging gradients of VDE and AEA in Figure 5 reflect the spectral broadening of the photoelectron peaks with increasing *n* and is consistent with an increase in λ for larger cluster sizes as the solvent reorganization naturally increases. Note that we are not aware of any other experimental method by which these fundamental physical properties can be determined for U^–^_(aq)_. While liquid microjet photoelectron spectroscopy can determine VDE_(aq)_, AEA_(aq)_, and λ,^52^ it is not clear how these methods can be applied to unstable radical anions such as U^–^_(aq)_.

From the perspective of aqueous uracil and within linear response theory,^53^ as illustrated in Figure 6, the position of each resonance relative to S_0_ in the neutral geometry and hydration sphere is ∼2λ (i.e., ∼2.4 eV in the present case) higher in the neutral geometry than the anion geometry. Hence, the π_3_* resonance of U_(aq)_ will be able to capture electrons with a vertical attachment energy, VAE_(aq)_ ∼ 1.6 eV (see Supporting Information). Similarly, the high-energy edge of the π_2_* resonance shifts to be above S_0_ in the neutral geometry so that it too is a resonance, which can be populated by electrons with very low-energy (VAE_(aq)_ ≲ 0.4 eV, see Supporting Information). Upon formation of either resonance, solvation will set in very rapidly (on a timescale less than 100 fs)^54,23^ to accommodate the change in charge state of the nucleobase, which will dynamically shift the resonance to lower energy. This would enable the π_2_* and π_3_* resonances to adiabatically evolve into bound states. While the resonances are unbound in the present cluster studies, internal conversion can clearly take place to form the bound anion ground state (as evidenced by thermionic emission, see Figure 3c). Such internal conversion to the anion ground state is likely to also be possible for the π_2_* and π_3_* states in solution once they have become vertically bound by solvent dynamics. Note also that the π_2_* state could participate in the electron capture mechanism of the π_3_* resonance by serving as an intermediate in the internal conversion from higher lying resonances, as has been observed in isolated molecules.^14,35^

Uracil is very similar to the other pyrimidine nucleobases, with both thymine (T) and cytosine (C) having similar electronic resonances in the gas-phase.^4^ Assuming that hydration has a similar overall stabilization and effect on the resonances, the pyrimidine π_2_* and π_3_* resonances of C and T are likely to be able to accept low electron energies, similar to U. The purine bases adenine and guanine have shape resonances that are generally at lower energy.^4^ Additionally, all nucleobases have core-excited (Feshbach) resonances at energies >4 eV,^21^ although these would likely have a lower capture probability. Nevertheless, some evidence for such a resonance is seen in Figures 1 and 4 at *hv* > 5 eV.

Our findings are broadly consistent with those from Kočišek et al. who showed that neutral U(H_2_O)_*m*_ clusters attached electrons over a relatively narrow range of <2 eV, which subsequently leads to evaporation of H_2_O molecules.^12^ This could be consistent with excitation to the π_2_* resonance, however, as their experiments have little control over cluster size, drawing direct comparisons is not possible. No other resonances were observed up to 3 eV electron energy. From Figure 5 and accounting for the reorganization energy, the π_3_* resonance is likely to lie at higher energy for small clusters (>3 eV, relative to the neutral ground state), and would therefore not be seen by their experiments. Moreover, signatures of the π_3_* resonance may also not be visible if thermionic emission dominates over evaporation at the higher electron energies.

Electron capture serves as a first step in the most commonly-adopted model leading to strand-breaks in DNA: the nucleobase accepts an electron in a π* orbital, which undergoes a nonadiabatic transition with the mostly repulsive σ* anion potential energy surface on the sugar-phosphate C–O bond that subsequently breaks.^55−57^ The π* resonances considered here could therefore serve as the entrance channel that triggers backbone fragmentation in aqueous DNA. Indeed, a clear and surprisingly narrow peak centered at ∼0.6 eV is observed in the yield of single-strand breaks of deposited DNA containing its structural water^58^ and a similar, though less pronounced peak, at very low energy to DNA in cells.^3^ Moreover, recent transient ultrafast radiology experiments have shown that radical anions are formed by low energy electron attachment to the nucleobases and nucleotides in aqueous solution.^59^ Hence, our gas-phase measurements appear to link up with observations in the bulk and potentially in biological environments, demonstrating the value of the bottom-up approach and 2D photoelectron spectroscopy as a tool to probe resonances in complex environments.

## Conclusions

The electron capture resonances of uracil in a water cluster environment have been determined by 2D photoelectron spectroscopy of the mass-selected anionic U^–^(H_2_O)_*n*_ clusters. Both the π_2_* and π_3_* shape resonances, which are well-known from electron transmission spectroscopy of isolated U, have been observed in the water clusters. A significant fraction of these resonances decay to form the ground state anion in all clusters studied (up to *n* = 10). Extrapolation with increasing U^–^(H_2_O)_*n*_ cluster size of the resonances, adiabatic detachment, and vertical detachment energies, offers a picture of the energetics and available resonances in a bulk aqueous environment. We find that AEA_(aq)_ = 3.53 ± 0.20 eV and VDE_(aq)_ = 4.75 ± 0.20 eV and that the reorganization energy, λ ∼ 1.2 eV. The broad π_2_* resonance in isolated uracil is a bound state in the anion geometry, but the high-energy edge remains a resonance in the neutral geometry. As a result, the π_2_* resonance can capture electrons with vertical attachment energies, VAE ≲ 0.4 eV, and rapid adiabatic hydration dynamics drives the electron binding. The π_3_* resonance with VAE_(aq)_ ∼ 1.6 eV remains unbound along a larger fraction of the reorganization coordinate, but eventually does become bound by 0.85 eV in the anion geometry. Recovery of the ground electronic state of the uracil anion is observed in all studied hydrated clusters, suggesting that even when the π_3_* is a resonance, internal conversion can compete to form a stable uracil anion. Hence, both the π_2_* and π_3_* resonances can capture electrons in the bulk aqueous environment leading to the radical uracil anion in its ground electronic state.

Our results provide a new route to explore electron impact resonances in complex environments. For uracil, we have determined the character of the scattering channels (i.e., open or closed) for the two lowest shape resonances in a bulk aqueous environment, providing a critical step towards identifying the resonances involved in the electron capture of DNA mediated by the nucleobases.

## Supporting Information Available

Mass-spectrum of U^–^(H_2_O)_*n*_, fitting details of photoelectron spectra, derivation of VAE_(aq)_, consideration of the excluded volume, ξ, and of errors (PDF)

## Notes

The experimental data generated (raw photoelectron images) have been deposited and can be accessed at https://doi.org/10.5281/zenodo.7400251.
