# Supplementary material for: Low-Energy Shape Resonances of a Nucleobase in Water
Source: J Am Chem Soc. 2022 Dec 30;145(2):1319–26. doi: 10.1021/jacs.2c11440 (PMC9853861; doi:10.1021/jacs.2c11440)
Supplement: Supplementary file 1 — ja2c11440_si_001.pdf [file ja2c11440_si_001.pdf]

Supporting Information for:

Low-energy Shape Resonances of a Nucleobase in Water

*Graham A. Cooper, Connor J. Clarke, and Jan R. R. Verlet**

Department of Chemistry, Durham University, DH1 3LE, United Kingdom

*j.r.r.verlet@durham.ac.uk

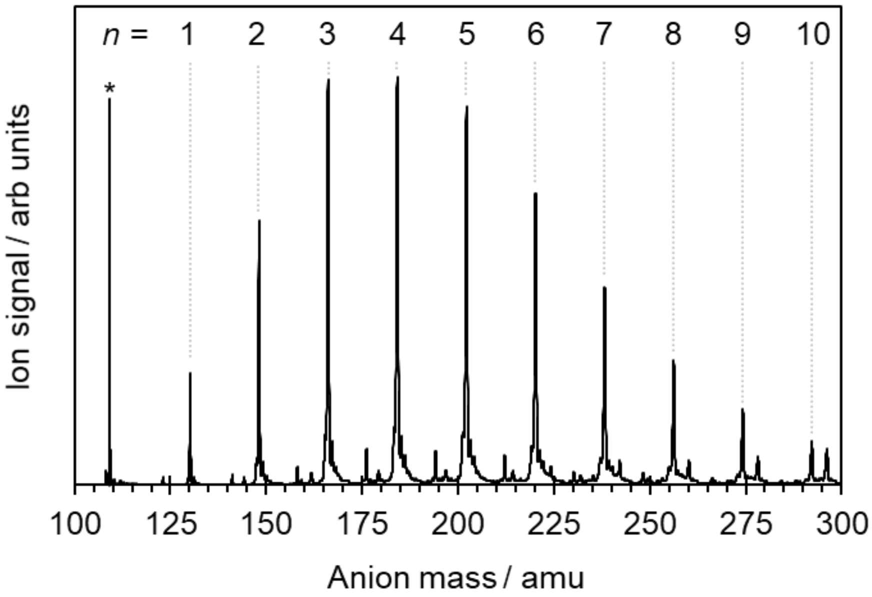

Figure S1: Representative mass spectrum for $U^{-}(H_2O)_n$. Peak highlighted with asterisk arises from electrons liberated by the laser used and is not part of the mass spectrum.

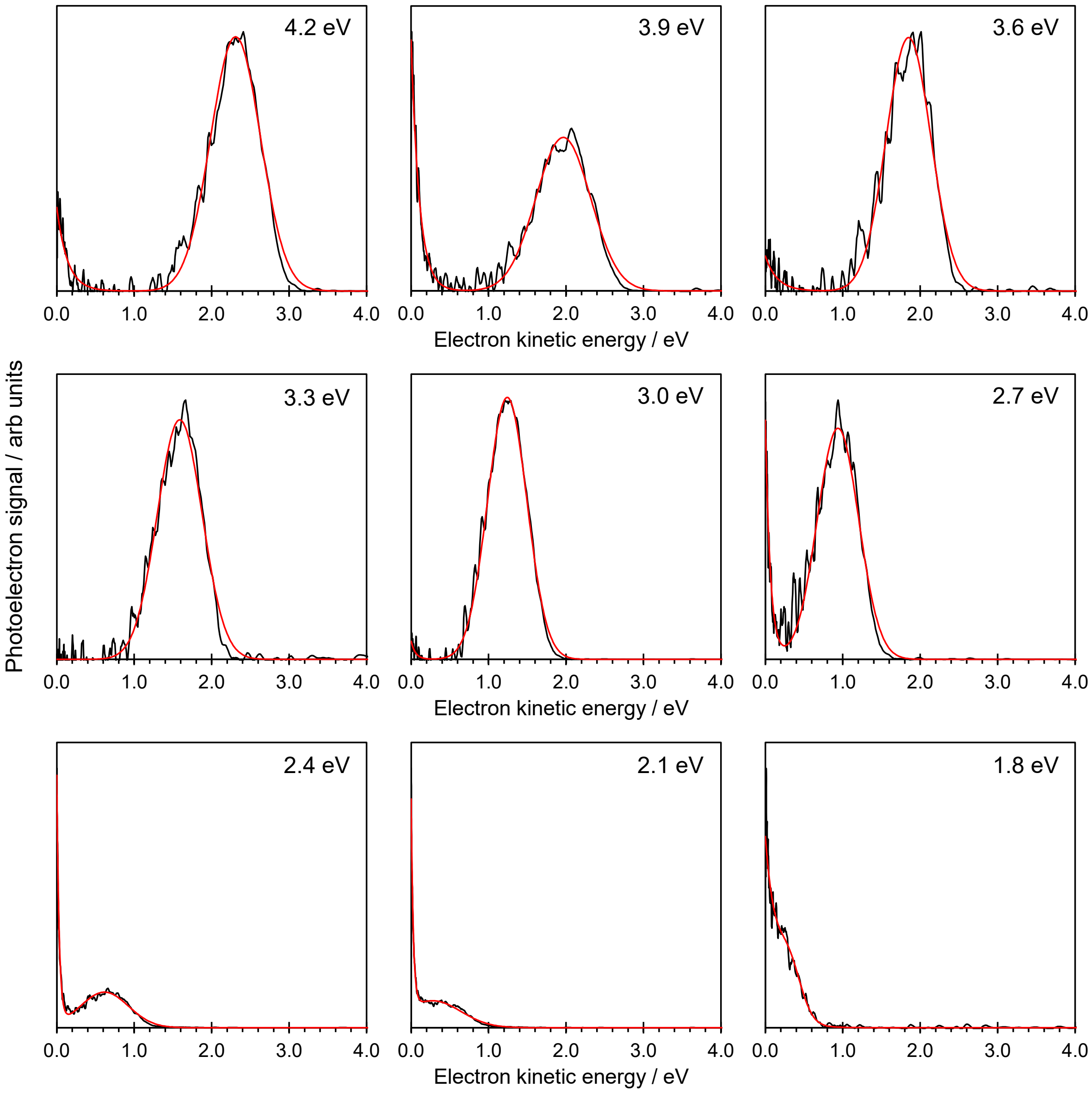

Figure S2: Representative photoelectron spectra (black line) for $U^{-}(H_2O)_4$ taken at a range of *hv*. Also included is a fit based on a sum of a Gaussian to represent the high-energy peak and an exponential decay to represent thermionic emission (red line). The amplitudes of the Gaussian and exponential can be seen to vary across *hv* with clear maxima in relative intensity of the exponential function when $hv < 2.7$ eV and at $hv \sim 3.9$ eV. The ratio of intensities of the amplitude of the exponential to that of the Gaussian is plotted in Figure 3 of the main manuscript.

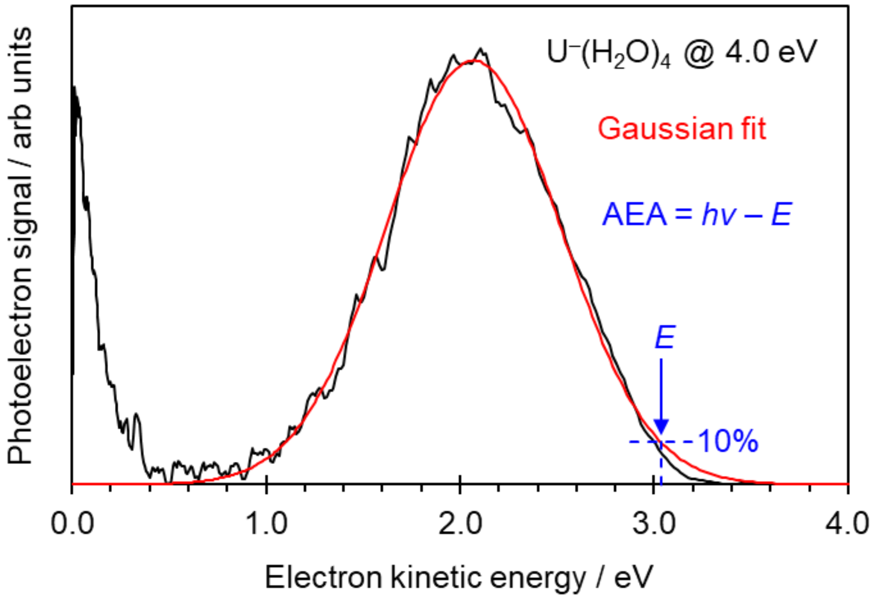

Figure S3: Representative fit of the non-resonant photoelectron spectrum (black line) to a Gaussian function (red line) for $U^{-}(H_2O)_4$ taken at $h\nu$ = 4.0 eV The vertical detachment energy, VDE, corresponds to the maximum of the peak. The adiabatic electron affinity, AEA, is also indicated and is taken as the value where the photoelectron signal reaches 10% of the maximum.

Effect and choice of ξ

The vertical detachment energy (VDE) of $U^{-}(H_2O)_n$ increases with cluster size. The corresponding gradient in the bulk limit should agree with that predicted by continuum dielectric theory,[1] which is independent of solute ion. Using expressions within the dielectric sphere approach detailed by Coe,[2] we determine a bulk-limit VDE vs $(n + \xi)^{-1/3}$ gradient of 6.00 eV, for our estimated cluster temperature of 100 K. This value is relatively insensitive to temperature (*e.g.* the gradient is 5.90 eV at room temperature).

Table S1: Extrapolated properties of $U^{-}_{(aq)}$ using different values of ξ. The gradient is calculated via a linear fit to the experimental VDE ($3 \leq n \leq 35$) plotted against $(n + \xi)^{-1/3}$. The highlighted value is that used in Figure 5.

ξ	VDE gradient / eV	$VDE_{(aq)}$ / eV	$ADE_{(aq)}$ / eV	λ / eV
0	3.63	4.02	2.98	1.04
3	5.39	4.58	3.40	1.18
4	5.95	4.75	3.53	1.22
5	6.50	4.91	3.65	1.26

Table S1 shows the effect ξ has on relevant experimental properties in the bulk limit. $\xi = 0$ ignores the volume of the uracil anion, giving a VDE vs $(n + \xi)^{-1/3}$ gradient in poor agreement with the dielectric sphere model. $\xi = 4$ reproduces the expected gradient very well, increasing the extrapolated VDE and adiabatic detachment energy (ADE). These energies change by less than 0.2 eV when setting $\xi = 3$ or $\xi = 5$. The reorganization energy is fairly insensitive to these changes.

ξ represents the volume ratio of the uracil anion to a single water molecule. Although our choice of ξ was guided by agreement with continuum dielectric theory, this parameter can also be estimated through its definition, providing additional justification for choosing $\xi = 4$. At room temperature, the partial molar volume of aqueous uracil is $V_U = 72$ cm^3 mol^{-1},[3] corresponding to $\xi = 4$. However, V_U refers to a volume change associated with hydration of the *neutral* uracil molecule – the anionic form of uracil occupies slightly less volume in solution due to electrostriction. Despite this, the partial molar volumes of neutral adenine (also a nucleobase)

and the deprotonated adenine anion are very similar (89.9 and 89.2 cm^3 mol^{-1}, respectively, at room temperature).[4] Therefore, using the neutral value is likely to offer a reasonable estimate of the molar volume of the anion.

The volume of the uracil anion can also be estimated using electronic structure calculations. The molecular volume can be defined as the volume within a contour of 0.001 electrons/$Bohr^3$ density. Density functional theory with the CAM-B3LYP functional[5] and the aug-cc-pVDZ basis set[6] obtains a molecular volume of 88 cm^3 mol^{-1} for the isolated uracil anion. Note that the limited diffuseness of this basis set ensures that the dipole-bound state of uracil is not considered. The corresponding value of ξ is slightly below 5. However, this procedure will overestimate the volume of the micro-hydrated uracil anion, as the surrounding water molecules align to restrict the volume. Exploratory calculations were performed on $U^-(H_2O)_6$ at a similar level of theory, lowering the molecular volume of the uracil anion to approximately 60 cm^3 mol^{-1}, giving some indication of the magnitude of this restriction.

Collecting the above considerations together, $\xi = 4 \pm 1$ appears to be consistent with continuum dielectric theory, (exploratory) electronic structure calculations, and measured thermodynamic properties. We therefore use this value and take the error as a reasonable and generous range. From Table S1, we therefore find an error in $VDE_{(aq)}$ and $ADE_{(aq)}$ of approximately ± 0.2 eV.

Derivation of aqueous values in Marcus picture (Figure 6)

From Figure 5 in main manuscript, we obtain the following values:

Vertical detachment energy of $U^{-}_{(aq)}$:	$VDE_{(aq)} = 4.75$ eV
Adiabatic electron affinity of $U^{-}_{(aq)}$:	$AEA_{(aq)} = 3.53$ eV
Reorganisation energy:	$\lambda = VDE_{(aq)} - AEA_{(aq)} = 1.22$ eV
Vertical excitation to π_2* of $U^{-}_{(aq)}$:	$VEE_{(aq)} \approx 1.5 - 2.7$ eV
Vertical excitation to π_3* of $U^{-}_{(aq)}$:	$VEE_{(aq)} = 3.89$ eV

In going from anion to neutral geometry, the energy increases by λ along reorganisation coordinate of the resonance and decreases by λ along the reorganisation coordinate of the resonance neutral ground state (S_0). Hence the energy chance from anion to neutral is 2λ = 2.44 eV. The vertical attachment energy (i.e. location of resonance in the neutral geometry) is then equal to the binding energy of the resonance in the anion geometry (a negative number if bound) plus twice the reorganisation energy. The binding energy of the resonance is given as $VEE_{(aq)} - VDE_{(aq)}$.

For the π_2* resonance, the highest energy edge has $VEE_{(aq)} \sim 2.7$ eV so that:

$$VAE_{(aq)} = VEE_{(aq)} - VDE_{(aq)} + 2\lambda \approx 2.7 - 4.75 + 2.44 \approx 0.4 \text{ eV}$$

For the π_2* resonance, the lowest energy edge has $VEE_{(aq)} \sim 1.5$ eV so that:

$$VAE_{(aq)} = VEE_{(aq)} - VDE_{(aq)} + 2\lambda \approx 1.5 - 4.75 + 2.44 \approx -0.8 \text{ eV}$$

For the π_3* resonance, the highest energy edge has $VEE_{(aq)} = 3.89$ eV so that:

$$VAE_{(aq)} = VEE_{(aq)} - VDE_{(aq)} + 2\lambda = 3.89 - 4.75 + 2.44 = 1.58 \text{ eV}$$

All relevant values are given in Figure S4

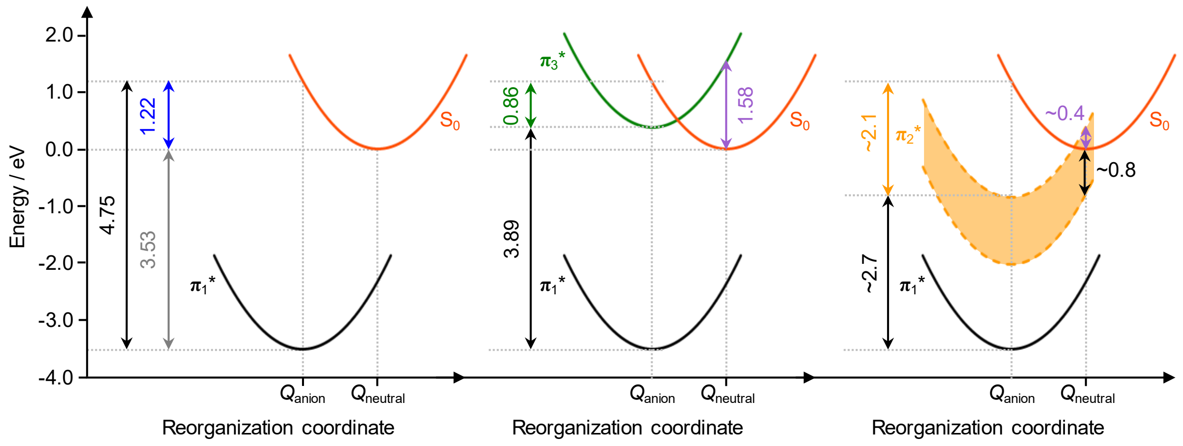

Figure S4: Marcus pictures with relevant aqueous energy values determined from the data in the main manuscript.

References

(1) Marcus, R. A. On the Theory of Oxidation-Reduction Reactions Involving Electron Transfer. I. *J. Chem. Phys.* **1956**, *24* (5), 966–978. https://doi.org/10.1063/1.1742723.

(2) Coe, J. V. Connecting Cluster Anion Properties to Bulk: Ion Solvation Free Energy Trends with Cluster Size and the Surface vs Internal Nature of Iodide in Water Clusters. *J. Phys. Chem. A* **1997**, *101* (11), 2055–2063. https://doi.org/10.1021/jp962490g.

(3) Fucaloro, A. F.; Dewey, K.; Fan, G.; Imuta, K.; Jensen, D.; Muranaka, M. Partial Molar Volumes of Uracil, Thymine, Adenine in Water and of Adenine in Aqueous Solutions of Uracil and Thymine. *J. Solut. Chem.* **2008**, *37* (9), 1289–1304. https://doi.org/10.1007/s10953-008-9302-2.

(4) Lowe, A. R.; Cox, J. S.; Tremaine, P. R. Thermodynamics of Aqueous Adenine: Standard Partial Molar Volumes and Heat Capacities of Adenine, Adeninium Chloride, and Sodium Adeninate from T=283.15K to 363.15K. *J. Chem. Thermodyn.* **2017**, *112*, 129–145. https://doi.org/10.1016/j.jct.2017.04.005.

(5) Yanai, T.; Tew, D. P.; Handy, N. C. A New Hybrid Exchange–Correlation Functional Using the Coulomb-Attenuating Method (CAM-B3LYP). *Chem. Phys. Lett.* **2004**, *393* (1), 51–57. https://doi.org/10.1016/j.cplett.2004.06.011.

(6) Peterson, K. A.; Woon, D. E.; Dunning, T. H. Benchmark Calculations with Correlated Molecular Wave Functions. IV. The Classical Barrier Height of the H+H2→H2+H Reaction. *J. Chem. Phys.* **1994**, *100* (10), 7410–7415. https://doi.org/10.1063/1.466884.
